# A Conserved PHD Finger Protein and Endogenous RNAi Modulate Insulin Signaling in *Caenorhabditis elegans*

**DOI:** 10.1371/journal.pgen.1002299

**Published:** 2011-09-29

**Authors:** Andres R. Mansisidor, Germano Cecere, Sebastian Hoersch, Morten B. Jensen, Trupti Kawli, Lisa M. Kennedy, Violeta Chavez, Man-Wah Tan, Jason D. Lieb, Alla Grishok

**Affiliations:** 1 Department of Biochemistry and Molecular Biophysics, Columbia University, New York, New York, United States of America; 2 David H. Koch Institute for Integrative Cancer Research, Massachusetts Institute of Technology, Cambridge, Massachusetts, United States of America; 3 Bioinformatics Group, Max Delbrück Center for Molecular Medicine, Berlin, Germany; 4 Department of Biology, Carolina Center for Genome Sciences and Lineberger Comprehensive Cancer Center, The University of North Carolina at Chapel Hill, Chapel Hill, North Carolina, United States of America; 5 Department of Genetics, Stanford University, Stanford, California, United States of America; 6 Department of Genetics and Development, Columbia University, New York, New York, United States of America; 7 Department of Microbiology and Immunology, Stanford University School of Medicine, Stanford, California, United States of America; Massachusetts General Hospital and Havard Medical School, United States of America

## Abstract

Insulin signaling has a profound effect on longevity and the oxidative stress resistance of animals. Inhibition of insulin signaling results in the activation of DAF-16/FOXO and SKN-1/Nrf transcription factors and increased animal fitness. By studying the biological functions of the endogenous RNA interference factor RDE-4 and conserved PHD zinc finger protein ZFP-1 (AF10), which regulate overlapping sets of genes in *Caenorhabditis elegans*, we identified an important role for these factors in the negative modulation of transcription of the insulin/PI3 signaling-dependent kinase PDK-1. Consistently, increased expression of *pdk-1* in *zfp-1* and *rde-4* mutants contributed to their reduced lifespan and sensitivity to oxidative stress and pathogens due to the reduction in the expression of DAF-16 and SKN-1 targets. We found that the function of ZFP-1 in modulating *pdk-1* transcription was important for the extended lifespan of the *age-1(hx546)* reduction-of-function PI3 kinase mutant, since the lifespan of the *age-1; zfp-1* double mutant strain was significantly shorter compared to *age-1(hx546)*. We further demonstrate that overexpression of ZFP-1 caused an increased resistance to oxidative stress in a DAF-16–dependent manner. Our findings suggest that epigenetic regulation of key upstream signaling components in signal transduction pathways through chromatin and RNAi may have a large impact on the outcome of signaling and expression of numerous downstream genes.

## Author Summary

Reduced activity of the insulin-signaling pathway genes has been associated with a longer lifespan and increased resistance to oxidative stress in animals due to the activation of important transcription factors, which act as master regulators and affect large networks of genes. The ability to manipulate insulin signaling and reduce its activity may allow activation of oxidative-stress response programs in pathological conditions, such as neuronal degeneration, where oxidative stress plays a significant role. Here, we describe a new way of inhibiting insulin signaling that exists in the nematode *Caenorhabditis elegans*. We find that transcription of one of the insulin-signaling genes is inhibited by mechanisms involving chromatin and RNA interference, a silencing process that depends on short RNAs. We demonstrate that mutants deficient in RNA interference are more susceptible to stress due to increased insulin signaling and that increased dosage of a chromatin-binding protein repressing insulin signaling and promoting RNA interference leads to better survival of nematodes grown under oxidative stress conditions. Since there is a clear homolog of this chromatin-binding protein in mammals, it may also act to promote resistance to oxidative stress in human cells such as neurons.

## Introduction

The role of RNA interference (RNAi) in the silencing of transposons and other repetitive elements is well documented [1], [2], while the knowledge of its impact on endogenous genes and signaling pathways is limited. In this article we investigate the remarkable and similar effects of the *Caenorhabditis elegans* RNAi-promoting factors RNAi DEficient 4 (RDE-4) [3] and Zinc Finger Protein 1 (ZFP-1) on the expression of stress-related genes. We focus on the key gene regulated by RDE-4 and ZFP-1, *pdk-1*, which encodes 3-phosphoinositide-dependent kinase-1 (PDK-1) [4], a component of a conserved insulin-signaling pathway. We describe a functional connection between *zfp-1*, *rde-4* and insulin signaling in genetic epistasis experiments and demonstrate the significance of *pdk-1* regulation by *zfp-1* and *rde-4* for *C. elegans* fitness.

ZFP-1, a Plant Homeo Domain (PHD) zinc finger protein, was first identified as a factor promoting RNAi interference in *C. elegans* [5]–[7]. It is a homolog of mammalian AF10 (Acute Lymphoblastic Leukemia 1-Fused gene from chromosome 10) [8] and plays a key role in leukemias caused by Mixed Lineage Leukemia MLL-AF10 fusion due to the recruitment of histone methyltransferase Dot1 by the AF10 portion of the fusion protein [9]. The developmental and physiological roles of AF10 are largely unknown. RDE-4 is a double-stranded RNA (dsRNA)-binding protein and a component of the Dicer complex responsible for the production of short interfering RNAs (siRNAs) from exogenous dsRNA [10]. The *rde-4(ne299)* null mutation was discovered in a screen for RNAi resistant mutants [3]. *rde-4(ne299)* does not have obvious developmental abnormalities, but shows synthetic phenotypes when combined with the null mutant in *C. elegans* Retinoblastoma gene *lin-35* [11] and appears to be less healthy at elevated temperatures [12]. Also, *rde-4* mutants were reported to have a slightly reduced lifespan [13]. The effects of *rde-4* loss-of-function are likely to be related to recently identified endogenous siRNAs (endo-siRNAs), which perfectly match thousands of genes in *C. elegans* either in sense or antisense orientation [14]–[17]. Indeed, the expression of some endo-siRNAs is diminished in the absence of *rde-4* [14], [18].

Our recent genome-wide mRNA expression study has revealed that ZFP-1 and RDE-4 affect the transcript levels of close to 250 overlapping genes [19]. Furthermore, putative target genes of endo-siRNAs [16] showed a significant enrichment among genes upregulated in the *rde-4(ne299)* null [3] and *zfp-1(ok554)* [20] loss-of-function mutant larvae [19]. We proposed that ZFP-1 and endo-siRNAs produced in an *rde-4*-dependent manner cooperate in the repression of target genes in the nucleus. Here, we confirm a direct repressive effect of ZFP-1 on endo-siRNA targets by comparing gene expression changes in *zfp-1(ok554)* and *rde-4(ne299)* with genome-wide localization of ZFP-1. Moreover, using functional analysis of misregulated genes we predict a role for RDE-4 and ZFP-1 in modulating insulin signaling and further demonstrate that regulation of *pdk-1* transcription by ZFP-1 and endogenous RNAi underlies the oxidative stress sensitivity and short lifespan of *zfp-1(ok554)* and *rde-4(ne299)* mutants.

## Results

### Gene expression signatures suggest a role for ZFP-1 and RDE-4 in modulating insulin signaling

In order to elucidate the common biological roles of ZFP-1 and endogenous RNAi we analyzed gene sets misregulated in *zfp-1(ok554)* and *rde-4(ne299)* mutants [19]. We found that genes with lowered expression in the mutants compared to the wild type were enriched in metabolic, oxidative stress-related and anti-pathogenic factors present in the intestine (Table S1). Since insulin signaling mutations lead to increased expression of factors important for defense against oxidative stress and pathogens [21]–[23], we decided to compare the lists of genes downregulated in *zfp-1(ok554)* and *rde-4(ne299)* with longevity-promoting “Class 1” genes found upregulated in the *daf-2* mutant in a *daf-16*-dependent manner [23].

**Figure 1:**
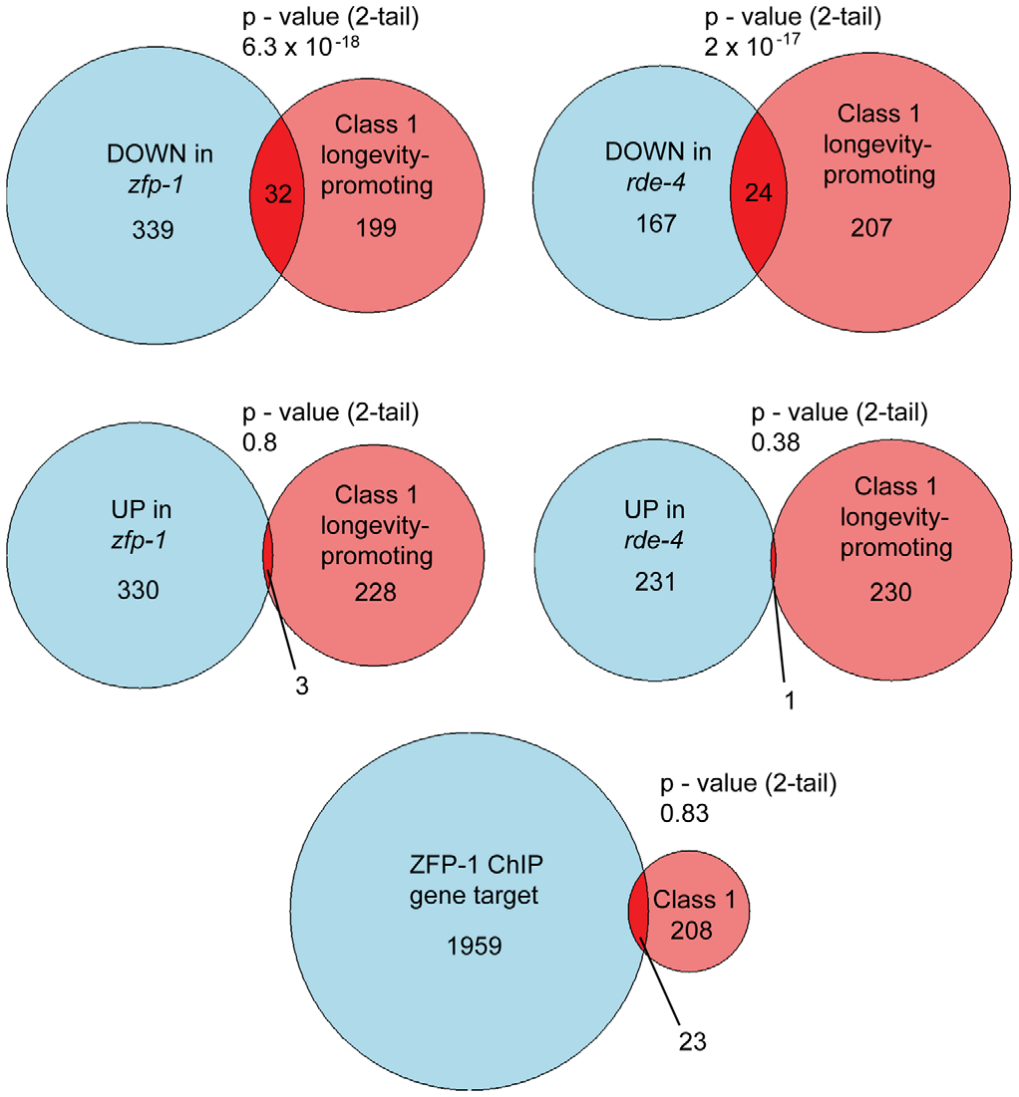
Longevity-promoting genes are expressed lower in *zfp-1(ok554)* and *rde-4(ne299)*. Venn diagrams are used to show overlaps between the “Class 1” longevity-promoting gene set from [23], gene sets determined to be UP- or DOWN-regulated in the mutants according to [19] and direct ZFP-1 target genes identified by ChIP/chip (modENCODE). Gene sets were first mapped to 18,459 genes with TOPOMAP [73] representation (with recalls ranging from 74% to 84%), and TOPOMAP-represented genes were included in the Venn diagrams. Fisher's exact test was used for calculating *p* values for overlaps.

**Figure 2:**
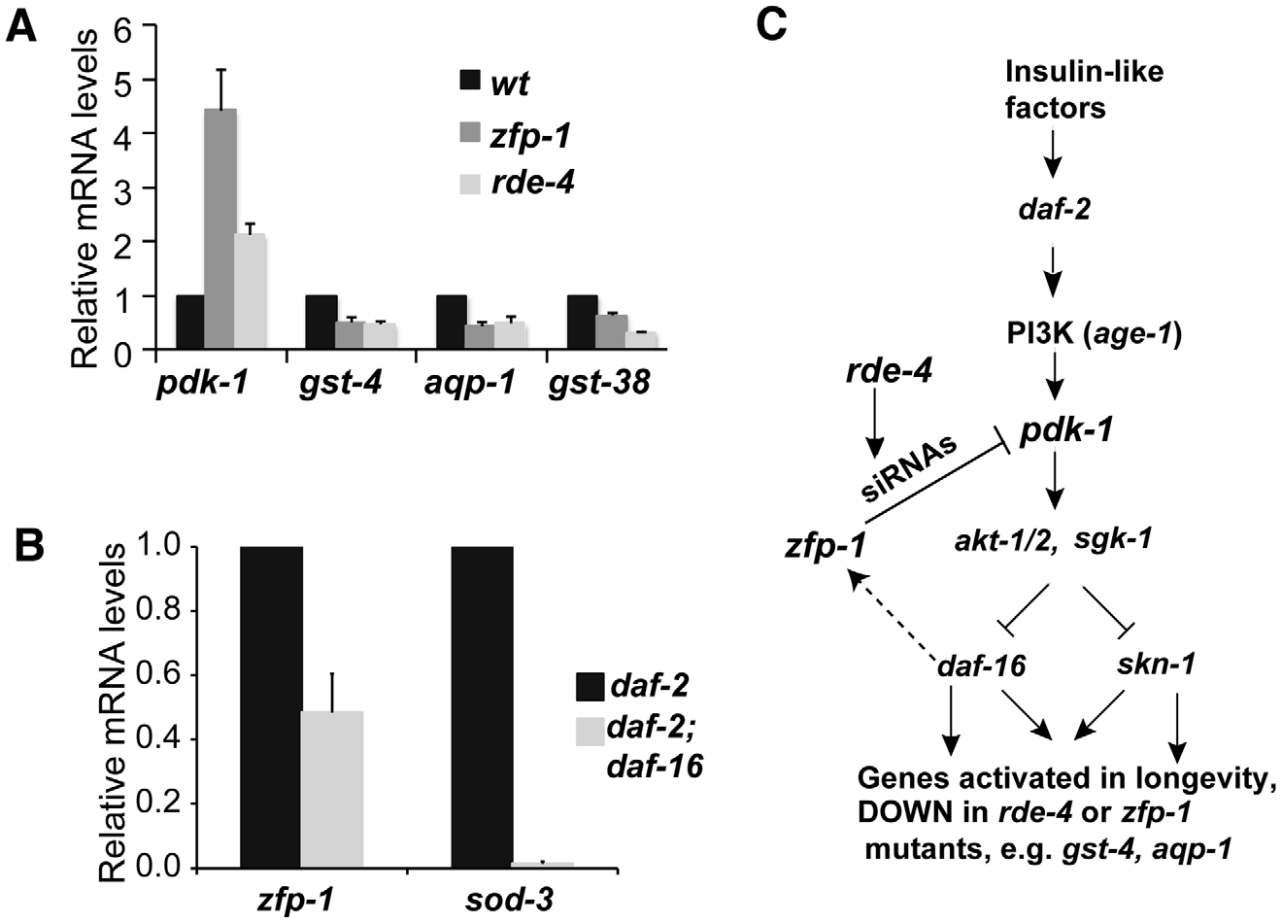
Gene expression signature connects *zfp-1*, *rde-4* and the insulin-signaling pathway. (A) *pdk-1* mRNA levels and mRNA levels of downstream targets repressed by insulin signaling as measured by real time RT-qPCR in the indicated mutants (L4 stage animals) and normalized to wild type. Results of three biological replicas are shown; error bars represent Standard deviation. (B) *zfp-1* and *sod-3* mRNA levels measured by real time RT-qPCR in *daf-2; daf-16* double mutant L4 worms and normalized to *daf-2* mutant background, results of two biological replicas are shown. (C) Insulin-signaling pathway in *C. elegans* modified according to results shown in (A, B) and Figure 1.

Insulin-like signaling in *C. elegans* via the DAF-2 insulin receptor and phosphatidylinositol 3-kinase (PI3K) negatively regulates the DAF-16/FOXO [24], [25] and SKN-1/Nrf [26] transcription factors. When insulin signaling is reduced, the enhanced DAF-16 and SKN-1 activities contribute to longer lifespan and stress resistance in worms due to concerted regulation of many of their targets [21]–[23], [27], [28]. DAF-16 and SKN-1 are negatively regulated in part at the level of their nuclear localization; therefore, mutants in this pathway are long-lived due to a higher level of the active nuclear DAF-16 and SKN-1 and appropriate transcriptional activation or repression of their direct targets. Our analyses revealed that genes downregulated in the *zfp-1* and *rde-4* mutants significantly overlapped with “Class 1” longevity promoting genes upregulated in the *daf-2* mutant (a condition when DAF-16 and SKN-1 are activated) [23] (Figure 1, Table 1, Table S1). Examples of genes whose expression is negatively regulated by daf-2 and positively regulated by *zfp-1* and/or *rde-4* include glutathione transferases *gst-4* and *gst-38*, and aquaporin (*aqp-1*) (Table 1, Figure 2A). Since RNAi is a gene-silencing phenomenon and gene sets expressed lower in *zfp-1(ok554*) and *rde-4(ne299*) are not enriched in endo-siRNA targets [19], we predict that “Class 1” longevity-promoting genes are regulated by ZFP-1 and RDE-4 indirectly. Consistently, genome-wide localization data showed no enrichment of ZFP-1 at longevity-promoting genes (Figure 1).

**Table 1:** Longevity-promoting genes [23] overlapping with genes expressed lower in *zfp-1(ok554)* L1-L2 larva according to [19].

Cosmid ID	Gene Name	Description	Mount^a^	Category^a^	Down in*rde-4* [19]
K12G11.3	*sodh1* */dod-11*	SOrbitol DeHydrogenase family	#08 Intestine		yes
C25E10.9	*swm-1*	Sperm activation Without Mating	#08 Intestine		no
C52E4.1	*cpr-1*	Cysteine Protease Related	#08 Intestine	Intestine	yes
F09F7.6		Protein of unknown function	#15		yes
F21C10.10		Protein of unknown function		Male enriched	yes
JC8.8	*ttr-51*	TransThyretin Related family domain	#22 Collagen		no
F48D6.4		Protein of unknown function	#08 Intestine		yes
PDB1.1		Mitochondrial Fe2+ transporter MMT1 and related transporters	#08 Intestine		no
F08B12.4		Protein of unknown function	#08 Intestine		yes
K01A2.2	*far-7*	Fatty Acid/Retinol binding protein	#15	Male enriched	yes
ZC395.5		Protein of unknown function	#15		yes
ZK1320.2		Protein of unknown function	#08 Intestine		yes
F54D5.3		Protein of unknown function	#08 Intestine		yes
K07C6.4	*cyp-35B1/* *dod-13*	Cytochrome P450 family	#22 Collagen	Cytochrome p450, lipid metabolism	no
W01B11.6		Protein of unknown function	#08 Intestine		no
F18E3.7	*ddo-2*	D-aspartate oxidase	#08 Intestine		no
F28A12.4		Aspartyl protease	#19 Amino acid metabolism	Proteases	no
F43H9.4		Protein of unknown function	#15		yes
C02A12.4	*lys-7*	LYSozyme	#08 Intestine		yes
F13D11.4		Flavonol reductase/cinnamoyl-CoA reductase	#14 Collagen		no
R12A1.4	*ges-1*	Abnormal Gut ESterase	#08 Intestine	Intestine	yes
R09B5.6	*hacd-1*	Hydroxy-Acyl-CoA Dehydrogenase	#22 Collagen	Biosynthesis; fatty acid oxidation; lipid metabolism	no
F46C5.1		Protein of unknown function	#08 Intestine		yes
C24B9.9	*dod-3*	Downstream of DAF-16	#15	Male enriched	yes
T19B10.2	*phi-59*		#14 Collagen		no
F09F7.7		2-Oxoglutarate- and iron-dependent dioxygenase-related proteins	#15		yes
Y43C5A.3		Protein of unknown function	#15		yes
ZK550.6		Peroxisomal phytanoyl-CoA hydroxylase	#08 Intestine		yes
F32A5.5	*aqp-1*	AQuaPorin	#08 Intestine		no
T22F3.11		Permease of the major facilitator superfamily	#08 Intestine		yes
T23G7.3		Telomerase elongation inhibitor/RNA maturation protein	#02 Germline enriched		no
B0218.8	*clec-52*	C-type LECtin	#08Intestine		no
K08F4.7^b^	*gst-4*	Glutathione S Transferase			yes
F35E8.8^b^	*gst-38*	Glutathione S Transferase	#24 Amino acid metabolism fatty acid oxidation; lipid metabolism		yes

**^a^**Functional annotation of genes is done based on TOPOMAP classification [73]

**^b^**These genes are prominently regulated by SKN-1 [74], *gst-38* is not listed among “class 1” longevity-promoting genes defined by [23].

### A higher level of *pdk-1* expression in *zfp-1(ok554)* and *rde-4(ne299)* correlates with lower expression of DAF-16 target genes

We considered the possibility that a direct target gene negatively regulated by *rde-4* and *zfp-1* would be de-repressed in the mutants to account for the reduced expression of the secondary targets, which may therefore be regulated by these factors indirectly. Indeed, a component of the insulin-signaling pathway, the kinase PDK-1, was among the most upregulated genes in *zfp-1* and *rde-4* [19] (Figure 2A). Although our microarray study was performed on *zfp-1* and *rde-4* mutant larvae (L1–L2), we found that *pdk-1* expression was increased in these mutants at other developmental stages as well (Figure 2A).

The *zfp-1* gene was shown to be a direct target of DAF-16 by chromatin immunoprecipitation (ChIP) combined with sequencing [29] and, more recently, using chromatin profiling by DNA adenine methyltransferase identification (DamID) [30]. However, it was not clear whether DAF-16 had a significant role in the regulation of *zfp-1*. We found that *zfp-1* mRNA expression in the *daf-2* mutant background was influenced by *daf-16* and was 2-fold lower in the *daf-2; daf-16* double mutant strain (Figure 2B). Therefore, DAF-16 appears to enhance transcription of *zfp-1*, although not nearly to the same extent as other prominent DAF-16 targets, such as *sod-3* (Figure 2B).

The analyses of gene expression described above suggest a model where ZFP-1 and RDE-4 modulate the insulin-signaling pathway by repressing *pdk-1* and that a DAF-16-dependent enhancement of *zfp-1* expression under conditions of low insulin signaling may contribute to a positive-feedback loop enhancing the effect of DAF-16 on other targets (Figure 2C).

### Nuclear localization of DAF-16::GFP conferred by the *pdk-1(sa709)* mutation persists in *zfp-1; pdk-1* and *rde-4; pdk-1* double mutants

**Figure 3:**
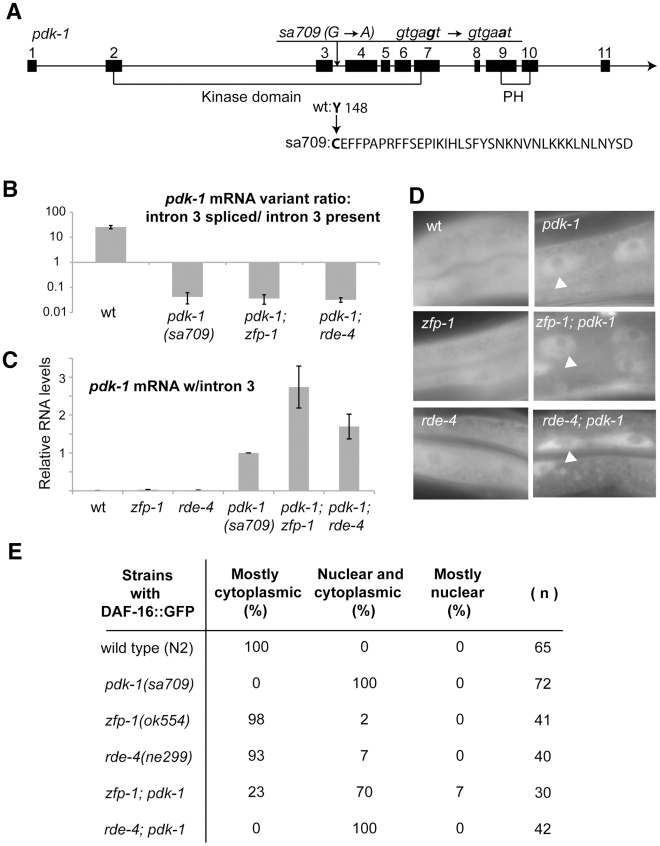
*pdk-1* loss-of-function mutation sa709 is epistatic to *zfp-1(ok554)* and *rde-4(ne299)*. (A) Schematic of the *pdk-1* gene with numbered boxes for exons and lines for introns, location of the sa709 mutation and predicted effects of the mutation on mRNA and protein; exons coding for the kinase domain and the pleckstrin homology domain (PH) according to [4] are indicated. (B) Expression levels of correctly spliced *pdk-1* mRNA and intron 3-containing mRNA were determined by RT-qPCR, and ratios of spliced/unspliced isoforms in the indicated mutants were calculated and presented on a graph. The forward primer used spanned the exon 1/exon 2 junction, the reverse primer for the spliced isoform spanned the exon 3/exon 4 junction and the reverse primer for the intron 3-containing isoform was intron 3-specific (see Materials and Methods). (C) Intron 3-containing *pdk-1* mRNA levels were measured by real time RT-qPCR in indicated mutants (L4 stage animals) and normalized to *pdk-1(sa709)*. Results of two biological replicas are shown; error bars represent Standard deviation. (D, E) DAF-16::GFP nuclear localization in indicated mutants was assessed as described in Materials and Methods. Representative epifluorescence images of intestinal cells (D) were taken on a Zeiss AxioImager Z1 microscope at 630x total magnification; white arrowheads point to the nuclei.

Next, we determined a molecular lesion in the weak loss-of-function *pdk-1* allele sa709 [4] and tested whether the *pdk-1(sa709)* mutant mRNA was still regulated by ZFP-1and RDE-4. We found that sa709 affects *pdk-1* mRNA splicing and leads to the incorporation of intron three into the mature *pdk-1* mRNA with a very low expression level of the correctly spliced mRNA in the mutant (Figure 3A, 3B). We combined *pdk-1(sa709)* with *zfp-1(ok554)* and found the level of mutant *pdk-1* mRNA expression to be elevated in the double mutant compared to *pdk-1(sa709)* alone (Figure 3C). The *pdk-1(sa709)* mRNA expression was also elevated in *rde-4(ne299); pdk-1(sa709)* (Figure 3C). Therefore, regulation of *pdk-1(sa709)* mRNA expression by ZFP-1 and RDE-4 was similar to that of wild type *pdk-1* mRNA.

Since loss-of-function mutations in insulin-signaling components lead to increased nuclear localization of DAF-16::GFP [31], we tested the *pdk-1(sa709)* allele in this assay and found that DAF-16::GFP had more prominent nuclear localization in *pdk-1(sa709)*, while it was mostly cytoplasmic in wild type, *zfp-1(ok554)* and *rde-4(ne299)* worms (Figure 3D, 3E). Nuclear localization of DAF-16::GFP persisted in *pdk-1; zfp-1* and *pdk-1; rde-4* double mutant animals (Figure 3D, 3E). These results demonstrate that *pdk-1(sa709)* is epistatic to *zfp-1(ok554)* and *rde-4(ne299)* and support a model where ZFP-1 and RDE-4 affect expression of DAF-16 targets through regulation of *pdk-1*.

### The short life span and enhanced sensitivity to oxidative stress of *zfp-1(ok554)* and *rde-4(ne299)* depend on PI3K signaling

**Figure 4:**
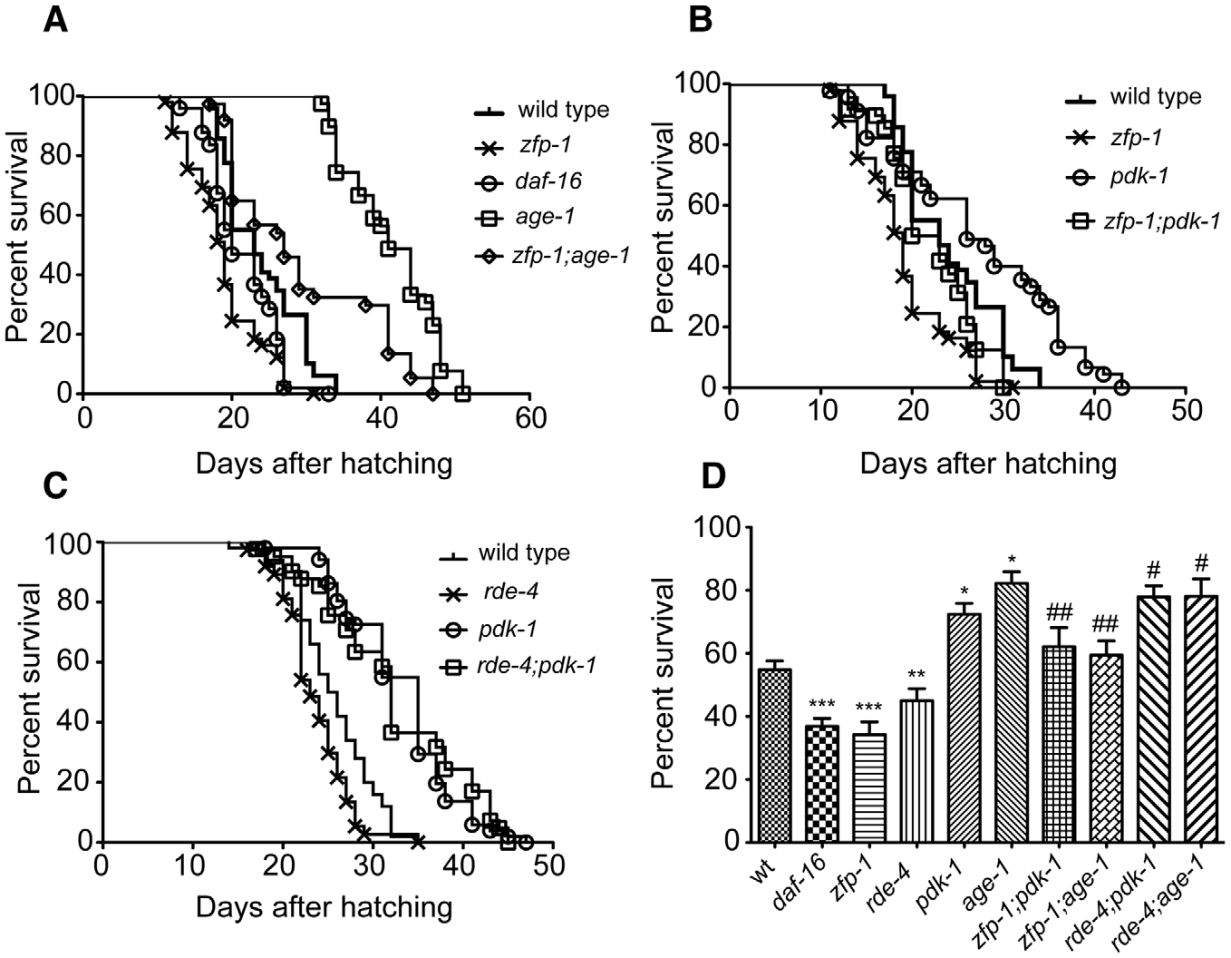
Functional connection between *zfp-1, rde-4* and insulin/PI3K signaling. (A-C) Life span of indicated mutant strains (see Materials and Methods). (A) Mean life spans were significantly different between wild type and all mutants (P<0.0001 *age-1*, P<0.01 *daf-16*, P<0.0001 *zfp-1*, P<0.001 *zfp-1; age-1*). Data shown is from one representative experiment that has been performed three times. (B) Mean life spans were significantly different between wild type and both *pdk*-*1* and *zfp-1* mutants (P<0.002 *pdk-1*, P<0.0001 *zfp-1*) while *zfp-1* was found to be significantly different from *zfp-1; pdk-1* (P<0.01). Data shown is from one representative experiment that has been performed three times. (C) Mean life spans were significantly different between wild type and all mutants (P<0.0001 *pdk-1*, P<0.01 *rde-4*, P<0.0001 *rde-4; pdk-1*). Data shown is from one representative experiment that has been performed two times. (D) Survival of L4 larva (n = 120) from indicated strains after 20 hour incubation period in 100mM paraquat: *** indicates significance of P<0.001, ** - P<0.01 and * - P<0.05 compared to wild type, ^#^ indicates significance of P<0.05 and ^##^ - P<0.01 compared to respective single mutant.

Since longevity-promoting genes have lower expression in the *zfp-1* and *rde-4* mutants, it is expected that they may live shorter than wild type worms. Indeed, a decrease in lifespan of *zfp-1(ok554)* [29] and *rde-4(ne299)* [13] has been reported, with the *zfp-1* mutant exhibiting a stronger phenotype than *rde-4*. In order to test whether upregulation of PDK-1 and therefore increased insulin signaling may contribute to the short lifespan of *zfp-1,* we conducted epistasis experiments with a reduction-of-function mutation in the PI3 kinase AGE-1, *age-1(hx546)* [32], [33]. We found that the short lifespan phenotype of *zfp-1(ok554)* was suppressed by *age-1(hx546)* (Figure 4A), i.e. the reduction in lifespan of the mutant was dependent on the active insulin signaling. Also, the extended lifespan of *age-1(hx546)* was dependent on ZFP-1 function, consistent with the possibility that enhanced PDK-1 dosage may suppress the defect in signaling conferred by the non-null *age-1* mutation (Figure 4A). Indeed, increased *pdk-1* dosage suppresses the constitutive dauer phenotype of *age-1(mg44)* [4].

In order to show that high levels of *pdk-1* expression contributed to the short lifespan of *zfp-1(ok554)* we attempted to combine *zfp-1(ok554)* with a strong loss-of-function mutation *pdk-1(sa680)* [4] for genetic suppression analyses. We were not able to recover *zfp-1(ok554); pdk-1(sa680)* and assume that this double mutant is not viable. Therefore, all epistasis analyses described below were performed with the sa709 allele.

*zfp-1* and *rde-4* affect expression of multiple target genes, and some phenotypes of *zfp-1(ok554)*, such as dauer promotion [29], are similar rather than opposite to the phenotypes of insulin-signaling mutants. We have found that *zfp-1; pdk-1* and *rde-4; pdk-1* double mutants display some egg-laying deficiency, which complicates the longevity assays that we conduct in the absence of drugs inducing sterility. However, although *zfp-1; pdk-1* and *rde-4; pdk-1* worms were undoubtedly sicker than *zfp-1* or *rde-4* single mutants, we found that the reduction of *pdk-1* function significantly suppressed the decreased lifespans of *zfp-1(ok554)* and *rde-4(ne299)* (Figure 4B, 4C). These results further support the idea that ZFP-1 and RDE-4 affect insulin signaling through the negative regulation of *pdk-1*.

The gene expression signatures of *zfp-1* and *rde-4* mutants suggested that they could be deficient in oxidative stress response. We induced oxidative stress by soaking L4 animals in 100mM paraquat and found that the *zfp-1(ok554)* mutant strain was much more sensitive to this treatment compared to the wild type (Figure 4D), similarly to *daf-16(mu86)* (Figure 4D), while *rde-4(ne299)* showed moderate sensitivity (Figure 4D, Figure S1A), and *age-1(hx546)* and *pdk-1(sa709)* were more resistant than wild type (Figure 4D). We found that *zfp-1; age-1*, *zfp-1; pdk-1* and *rde-4; age-1, rde-4; pdk-1* double mutants were less sensitive to oxidative stress than *zfp-1* and *rde-4*, respectively (Figure 4D), indicating that the stress sensitivity of *zfp-1(ok554)* and *rde-4(ne299)* was due to active insulin/PI3K signaling.

In order to determine whether increased *pdk-1* expression may be sufficient to cause a stress sensitivity phenotype, we tested the SP940 strain, which contains the free duplication mnDp (II;X;f) that includes the *pdk-1* locus. We found that *pdk-1* mRNA levels are increased about 2.5-fold in this strain (Figure S1C), close to that observed in *rde-4(ne299)*, and it shows a comparable sensitivity to paraquat (Figure S1A, S1B). These data are consistent with the idea that regulating *pdk-1* dosage is important for animal fitness.

### Increase in ZFP-1 expression promotes resistance to oxidative stress in a DAF-16–dependent manner

**Figure 5:**
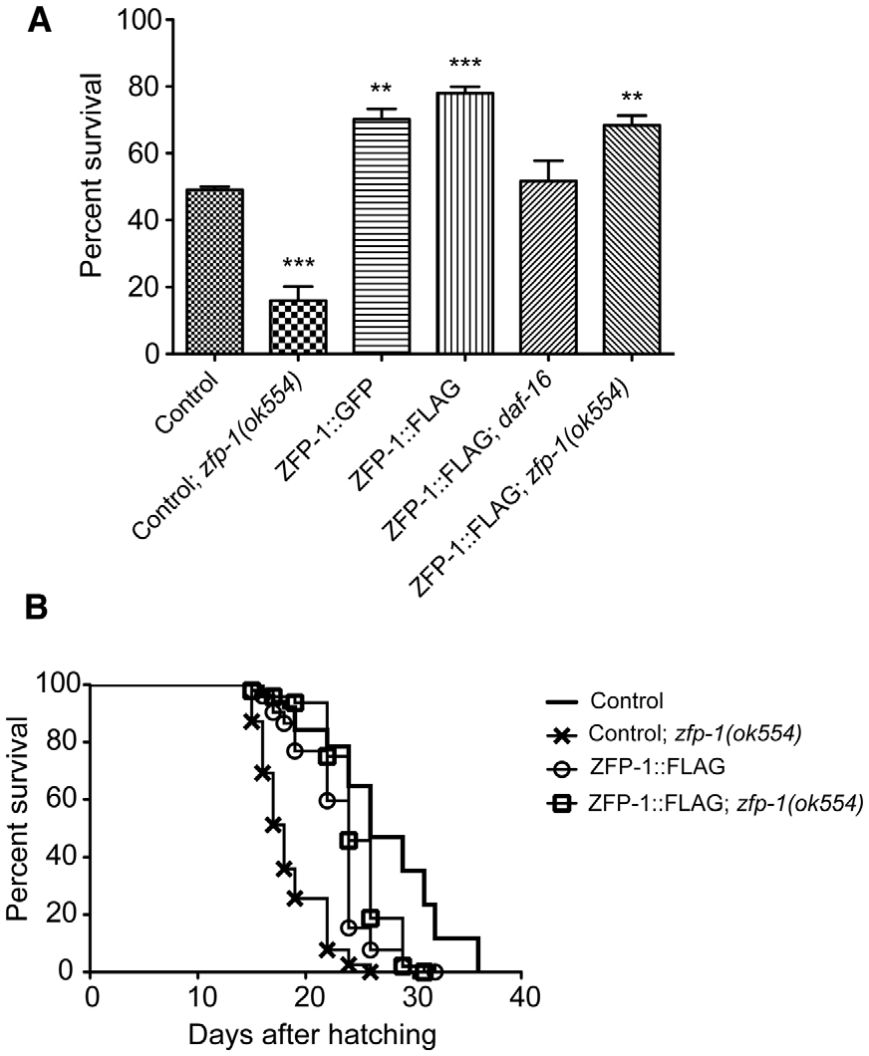
Increase in ZFP-1 expression promotes resistance to oxidative stress. (A) Survival of L4 larva (n = 120) from indicated strains after 20 hour incubation period in 100mM paraquat: *** indicates significance of P<0.001, ** - P<0.01 and * - P<0.05 compared to the control transgenic strain. (B) Life span of indicated transgenic strains. Mean life spans were significantly different between the control strain and all other strains (P<0.0001). Control; *zfp-1* was found to be significantly different from both ZFP-1::FLAG and ZFP-1::FLAG; *zfp-1* (P<0.0001). Life spans were determined in parallel for all strains; data shown is from one representative experiment that has been performed two times.

We generated transgenic lines expressing ZFP-1::GFP and ZFP-1::FLAG fusion proteins by introducing tags into the C-terminal region of ZFP-1 through fosmid recombineering in bacteria [34]. The resulting genes are expressed from the 30kb fosmid and are subject to the same regulatory inputs as the endogenous *zfp-1* locus; the transgenes fully rescued the stress sensitivity and reduced lifespan phenotypes of the *zfp-1* mutant (Figure 5A, 5B). *zfp-1* mRNA expression was about two-fold greater in ZFP-1 transgenic lines compared to the control (Figure S2). Moreover, we found that these ZFP-1 overexpressing lines were more resistant to oxidative stress compared to the control line generated by a similar technique of *unc-119* mutant rescue but not containing the ZFP-1 fosmid (Figure 5A). The stress resistance of ZFP-1 overexpressing lines was dependent on DAF-16 function (Figure 5A). This is consistent with the repression of insulin signaling and therefore indirect activation of DAF-16 by ZFP-1. We have not observed lifespan extension in the ZFP-1 overexpressing lines (Figure 5B), which indicates that a higher level of ZFP-1 may be advantageous only in acute stress situations.

### ZFP-1 functions to protect the animals against pathogenic challenge

**Figure 6:**
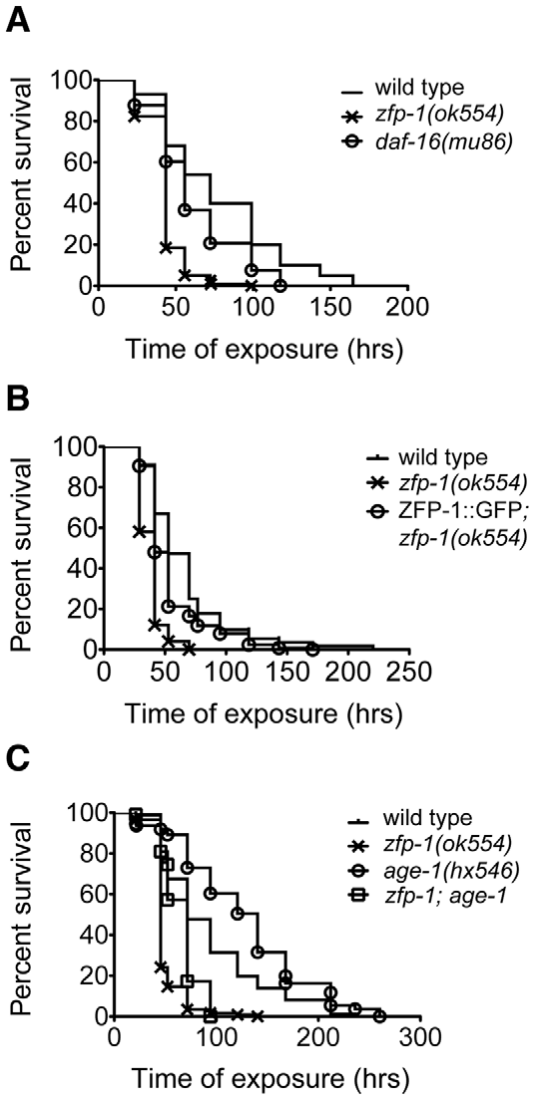
*zfp-1(ok554)* is sensitive to *P. aeruginosa.* (A–C) Killing assays demonstrating survival on *P. aeruginosa* (PA14). (A) Percent survival was found to be significantly different between wild type and both *daf-16* and *zfp-1* mutants (P<0.0001 *zfp-1*, P<0.0004 *daf-16*). (B) Percent survival was found to be significantly different between wild type and both *zfp-1(ok554)* and ZFP-1::GFP; *zfp-1* (P<0.0001 *zfp-1*, P<0.01 ZFP-1::GFP; *zfp-1*); *zfp-1(ok554)* was found to be significantly different from ZFP-1::GFP; *zfp-1* (P<0.0001). (C) Percent survival was significantly different between wild type and all mutants (P<0.0001 *age-1*, P<0.0001 *zfp-1*, P<0.001 *zfp-1; age-1*); *zfp-1(ok554)* was found to be significantly different from *zfp-1; age-1* (P<0.0001). Data shown is from one representative experiment that has been performed three times (see Materials and Methods).

An example of an acute stress response is the response of animals to pathogens. The human pathogenic bacterium *Pseudomonas aeruginosa* (PA14) inhibits DAF-16 nuclear localization and therefore downregulates the expression of defense factors that are dependent on DAF-16 [35]. We tested the effect of the loss of ZFP-1 function on innate immunity by assaying the survival of *zfp-1(ok554)* animals. Upon exposure to PA14 under the standard infection assay conditions [36], we observed that the *zfp-1(ok554)* mutants were significantly more susceptible to *P. aeruginosa* infection- mediated killing (Figure 6A, 6B). The pathogen sensitivity seen in *zfp-1(ok554)* mutants was due to loss of ZFP-1 function as was confirmed using a ZFP-1::GFP transgene that rescued the mutant phenotype (Figure 6B).

Next, we tested whether the increased susceptibility of *zfp-1(ok554)* to PA14 was dependent on insulin signaling. We confirmed that *age-1(hx546)* was more resistant to the infection (Figure 6C) and tested *age-1; zfp-1* double mutants. The results were similar to those found in the longevity assays: *age-1* and *zfp-1* suppressed each other's phenotypes (Figure 6C). The survival of the double mutant was closer to that of *zfp-1(ok554)* than *age-1(hx546)*, although *age-1* significantly suppressed the sensitivity of *zfp-1* to PA14 killing. We conclude that PI3K signaling contributes to the pathogen-sensitivity of *zfp-1(ok554).*

### ZFP-1 localizes to the *pdk-1* promoter but not to the promoters of DAF-16 targets

**Figure 7:**
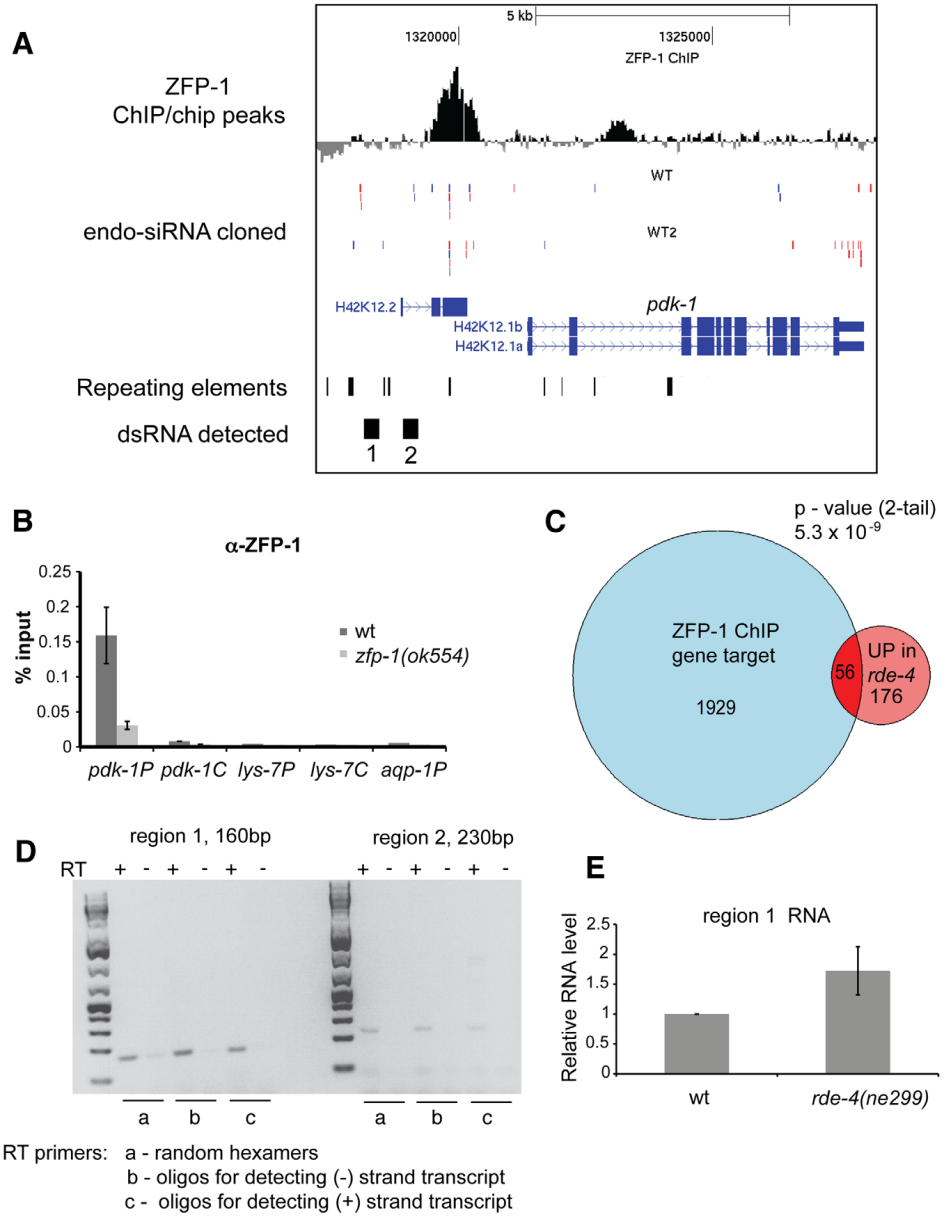
ZFP-1, dsRNA, and siRNAs localize to the promoter of *pdk-1*. (A) A screen shot of the *pdk-1* locus generated using the UCSC browser and indicating ZFP-1 localization peaks (ChIP/chip modENCODE data), cloned endo-siRNAs from [38] - (WT1) and [37] - (WT2), and promoter regions 1 and 2 with detected bi-directional transcription shown in (D). Antisense siRNAs are indicated in red, sense siRNAs in blue. (B) ZFP-1 ChIP/PCR with antibodies recognizing the C-terminus of the protein used for ChIP/chip shown in (A) in wild type and *zfp-1(ok554)* demonstrating DNA enrichment in IP relative to input by qPCR on the promoters – “P” and coding regions – “C” of the genes shown. Results of two biological replicas are shown; error bars represent Standard deviation. (C) Venn diagram showing statistically significant overlap between ZFP-1 target genes identified by ChIP/chip, where the ZFP-1 peak was found in the 1,500bp promoter window, and genes upregulated in *rde-4(ne299)* from [19]. (D) RT-PCR detecting transcription from both (−) and (+) DNA strands at indicated regions of the *pdk-1* promoter. (E) RT-qPCR measuring expression from the region 1 in the *pdk-1* promoter. The average of four biological replicas is shown, error bars represent Standard deviation, P<0.05.

**Figure 8:**
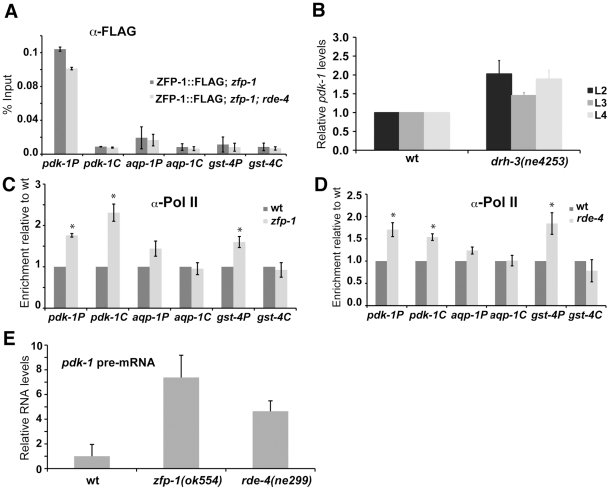
ZFP-1 and RDE-4 regulate transcription of *pdk-1*. (A) ZFP-1::FLAG ChIP with anti-FLAG antibodies in wild type and *rde-4(ne299)* demonstrating DNA enrichment in IP relative to input by qPCR on the promoters – “P” and coding regions – “C” of the genes shown. Results of two biological replicas are shown; error bars represent Standard deviation. (B) *pdk-1* mRNA levels measured by RT-qPCR in the *drh-3* mutant at different larval stages and normalized to wild type. (C, D) RNA polymerase II ChIP with 8WG16 antibodies (Covance) in wild type and *zfp-1(ok554)* (C) or wild type and *rde-4(ne299)* (D) demonstrating DNA enrichment relative to wild type by qPCR on the promoters – “P” and coding regions – “C” of the genes shown. Results of two biological replicas are shown; error bars represent Standard deviation; * indicates significance of P<0.05 compared to wild type. Worm preps used for Pol II ChIP were the same as those used for mRNA expression analysis shown in Figure 2A. (E) *pdk-1* pre-mRNA levels measured by RT-qPCR in indicated mutants (L4 stage animals) and normalized to wild type. Results of two biological replicas are shown; error bars represent Standard deviation.

Consistent with our expression and genetic epistasis data suggesting a direct role of ZFP-1 in repressing *pdk-1* transcription, a strong peak of ZFP-1 localization was found at the *pdk-1* promoter in ChIP/chip experiments conducted by the modENCODE (model organism ENCyclopedia Of DNA Elements) project (Figure 7A). We confirmed ZFP-1 localization to the *pdk-1* promoter by ChIP/PCR with antibodies specific to endogenous ZFP-1 (Figure 7B) as well as with anti-FLAG antibodies in experiments with ZFP-1::FLAG transgenic lines (Figure 8A).

ZFP-1 was not localized to the promoters of other genes of the insulin signaling pathway (*daf-2*, *age-1*, *akt-1*, *sgk-1*); it was also not present at DAF-16 target genes that have reduced expression in *zfp-1(ok554)* and appear to be positively regulated by this factor, as discussed earlier (Figure 1, Figure 7B, Figure 8A, and Table S1). There was no enrichment in direct ZFP-1 targets among the longevity-promoting genes (P-value 0.83) (Figure 1). Therefore, ZFP-1 is likely to target directly only genes whose transcription it inhibits (Table S1).

### Endogenous siRNAs map to the *pdk-1* promoter

We reported previously a very significant overlap between genes negatively regulated by *zfp-1* and *rde-4* and endogenous siRNA target genes [19]. Consistently, we find that direct ZFP-1 target genes are overrepresented among genes expressed higher in the *rde-4* mutant (Figure 7C). *pdk-1* is repressed by ZFP-1 and is also negatively regulated by RDE-4, which is a dsRNA-binding protein required for the biogenesis of siRNAs in the exogenous RNAi pathway [10] and contributing to the biogenesis of some endo-siRNAs [14], [18]. Knowing this, we searched available deep sequencing data [37]–[40] for endo-siRNAs mapping to the *pdk-1* locus. There were few endo-siRNAs corresponding to the coding region of *pdk-1*, and more siRNAs mapped to the promoter region of the gene (5kb upstream of the transcription start site), including a predicted open reading frame, H42K12.2. (Figure 7A). However, for this open reading frame, no transcriptional evidence exists, neither from EST collections nor from deep sequencing runs undertaken in the context of the modENCODE project, and it therefore appears to be a mis-annotated gene [41], [42]. We were able to detect ∼100–250 nt transcripts at the *pdk-1* promoter produced from both the plus and minus DNA strands, consistent with the possibility of dsRNA production and processing by RDE-4 and Dicer (Figure 7D and Text S1). Moreover, we detected an elevated level of this RNA in the *rde-4* mutant (Figure 7E), further supporting the possible involvement of RDE-4 in the dsRNA processing. Unfortunately, *pdk-1* promoter-specific endo-siRNAs are expressed at a very low level, and we were not able to reliably detect them by RT-qPCR. Nevertheless, additional evidence for *pdk-1* regulation by endogenous RNAi comes from the observation that *pdk-1* mRNA levels are increased in *drh-3(ne4253),* a loss-of-function mutant in *d*icer-*r*elated *h*elicase 3 [38], (Figure 8B). DRH-3 is thought to participate in multiple branches of endogenous RNAi in *C. elegans* [38].

### RNA polymerase II occupancy at the *pdk-1* coding region is increased in *zfp-1(ok554)* and *rde-4(ne299)*

We have shown that both *zfp-1* and *rde-4* affect the longevity of *C. elegans* and its ability to resist oxidative stress and that *pdk-1* mRNA levels are elevated in *zfp-1(ok554*) and *rde-4(ne299*) ([19] and Figure 1 and Figure 3). Furthermore, we have demonstrated that ZFP-1 binds the *pdk-1* promoter and that endogenous siRNAs also have a potential to regulate *pdk-1* directly. Next, we analyzed RNA polymerase II (Pol II) occupancy at the *pdk-1* promoter and coding region by ChIP in wild type, *zfp-1(ok554*) and *rde-4(ne299*) L3-L4 animals and found it to be significantly increased in both mutants (Figure 8C, 8D). Consistent with transcriptional regulation, *pdk-1* pre-mRNA levels were elevated in both mutants as well (Figure 8E). RDE-4, and therefore *rde-4-*dependent endo-siRNA production, did not affect ZFP-1 localization to the *pdk-1* promoter (Figure 8A). It is possible that ZFP-1 and the RNAi machinery are independently recruited to the same targets and cooperate in inhibiting their transcription. Alternatively, ZFP-1 may help stabilize downstream RNAi factors at the endo-siRNA target genes.

Pol II levels increased only at the promoters, but not at the coding regions of indirect target genes expressed lower in *zfp-1(ok554*) and *rde-4(me299*) (Figure 8C, 8D), a signature consistent with a slower rate of transition from transcriptional initiation to elongation [43]. This finding reflects the lower expression of these genes in the mutants, although they are not regulated directly by ZFP-1 and do not belong to the group of prevalent endo-siRNA targets ([19] and Table S1).

We have previously described a very significant overlap between genes misregulated in *zfp-1(ok554*) and genes misregulated in *rde-4(ne299*) and noted that the level of expression of *zfp-1* mRNA did not change in *rde-4(ne299*) and *vice versa* [19]. Since the *rde-4* mutation has milder effects on gene expression than *zfp-1(ok554*), they could potentially be due to *zfp-1* misregulation. Therefore, we further confirmed that protein levels of ZFP-1 are not decreased in *rde-4(ne299*) (Figure S3).

ZFP-1 localizes to the *pdk-1* promoter and both the *pdk-1* mRNA level and Pol II occupancy at the *pdk-1* gene are increased in *zfp-1(ok554*). These findings strongly suggest that transcription of *pdk-1* is directly and negatively modulated by ZFP-1. Our genetic and molecular data also clearly demonstrate that rde-4 has a role in the transcriptional regulation of *pdk-1*. Several lines of evidence provide correlative support for a possible direct role of endo-siRNAs in *pdk-1* regulation: endo-siRNAs match the *pdk-1* promoter in a region also targeted by ZFP-1, dsRNA production is detected at the promoter and is increased in *rde-4(ne299*), and *pdk-1* mRNA levels are elevated in at least two RNAi pathway mutants. However, since the endo-siRNAs targeting *pdk-1* are not very abundant, we were not able to determine whether they change in *rde-4(ne299*), and there is a possibility that *rde-4* affects *pdk-1* transcription indirectly. In either case, RDE-4 is most likely involved in gene regulation through endo-siRNA production since this is the only known molecular function of this protein. The relatively more abundant endo-siRNAs matching the *pdk-1* promoter (Figure 7A) are not unique and correspond to the repeat sequences. The Argonaute proteins that bind endo-siRNAs and work downstream in the RNAi pathways have been described and include at least two separate branches: the CSR-1 branch [37] and the WAGO branch [38]. Although CSR-1-bound endo-siRNAs are enriched in sequences antisense to protein-coding genes, they also include endo-siRNAs matching repeats [37], while the WAGO system appears to preferentially target repeats and pseudogenes [38]. Both the WAGO and CSR-1 systems have been shown to have some connection to the RDE-4-regulated genes [38], [44], and ZFP-1 ChIP/chip targets are enriched in both WAGO and CSR-1-dependent endo-siRNA target gene sets (G. Cecere, M. Jensen, et al., manuscript in preparation). Therefore, we think that regulation of some endogenous genes, such as *pdk-1*, which contain simple repeats in their promoters, may have evolved to depend on the RNAi surveillance system, either WAGO or CSR-1-based.

## Discussion

### ZFP-1/AF10 and resistance to oxidative stress

This work has revealed a direct repression of *pdk-1* transcription by *C. elegans* AF10 homolog ZFP-1 and the significance of this transcriptional regulation in modulating insulin signaling. We have demonstrated that overexpression of ZFP-1 leads to enhanced resistance to oxidative stress in nematodes in a DAF-16-dependent manner. The role of DAF-16/FOXO in longevity and stress response is conserved in animals [45], and it would be interesting to see whether AF10 has a role in promoting stress resistance through the activation of FOXO. FOXO proteins have been shown to cause a neuroprotective effect in *C. elegans*, *Drosophila* and mammalian models of neurodegeneration [46]. Another transcription factor involved in the antioxidant response, Nrf2 – a homolog of *C. elegans* SKN-1 - has been implicated in the neuroprotection of motor neurons in a mouse model of ALS [47], while SKN-1 was shown to be important for protection of dopamine neurons against methylmercury-induced degeneration in *C. elegans* [48]. Since both DAF-16 and SKN-1 are negatively regulated by insulin/PI3K signaling in *C. elegans* [45] (Figure 2C), perhaps inhibition of this signaling pathway in mammalian neurons will lead to activation of both FOXO3a and Nrf2. Our work suggests that the homolog of ZFP-1, AF10, may have a neuroprotective effect by indirectly activating FOXO3a and Nrf2 if the regulation of *pdk-1* by ZFP-1/AF10 is conserved in animals.

### Endogenous RNAi in gene expression regulation

RNAi was discovered in *C. elegans* as a response to exogenously introduced dsRNA [3], [49] and was considered to be primarily an anti-viral mechanism also directed against repetitive elements [50], especially since the first RNAi-resistant mutants did not have obvious developmental phenotypes [3]. The discovery of mutants in RNA-dependent RNA polymerase (RdRP) genes that displayed developmental phenotypes [51], [52] and were either RNAi-resistant [51] or more sensitive to exogenous RNAi [52], highlighted the possibility that RNAi may be used for regulating endogenous genes. Indeed, endogenous siRNAs antisense to protein-coding genes and similar to those produced during exogenous RNAi were discovered first in the worm [15] and then in other animals [53]. It became apparent that in mutants lacking specific endo-siRNAs, corresponding mRNAs become upregulated [14], [54], [55], and microarray and deep sequencing approaches have been used for identifying genes that change expression in the RNAi mutants [13], [14], [19], [37]–[39], [44], [56]–[58]. However, the significance of misregulation of specific genes for the biology of the worm has not been clearly demonstrated and phenotypes described for RNAi-related mutants [13], [37], [51], [54]–[61] were not connected to specific targets by functional epistasis experiments. This study interprets the microarray signature of *zfp-1* and *rde-4* mutants, demonstrates short lifespan and stress sensitivity phenotypes consistent with the signature, and provides functional evidence that *pdk-1* is a major target responsible for these phenotypes through genetic epistasis, RNA expression and ChIP analyses.

RNAi in *C. elegans* has the potential to cause both post-transcriptional [49] and transcriptional [6], [62] gene silencing. It is possible that endogenous RNAi utilizes multiple mechanisms and that some genes are subject mostly to post-transcriptional regulation while others are regulated at the transcriptional level; the latter are likely to have fewer matching endo-siRNAs to the coding region and relatively more promoter-specific endo-siRNAs, like *pdk-1*. We surveyed the genes upregulated in *rde-4(ne299*) for an endo-siRNA signature similar to that of the *pdk-1* locus and found a number of examples (Figures S4 and S5). Interestingly, most of these types of genes, including *pdk-1*, have repetitive elements at the promoters and endo-siRNAs matching them. It appears that a modulating effect of RDE-4 on the transcription of some endogenous genes is linked to the control of repetitive elements.

RNAi-dependent silencing of long terminal repeats (LTR) and non-coding RNA genes located in euchromatic regions that functions with trace amounts siRNAs has been described recently in *S. pombe* [63]. The lack of abundant siRNA species was remarkable, considering that Dicer and RdRP interacted physically with the loci and that LTR transcript levels were significantly elevated in the *dcr1*, *ago1* and *rdp1* mutants. This type of RNAi-based regulation appears to be very similar to that operating on the *pdk-1* gene in *C. elegans* that we describe here.

Examples of genes regulated by RNAi through repetitive elements in promoters already exist in *Arabidopsis* and include the FWA gene, which affects flowering time [64], [65] and, more recently, an extracellular peroxidase *Ep5C* gene [66]. High levels of *Ep5C* promote susceptibility to *Pseudomonas syringae* and mutation in the Argonaute 4 gene was recovered in an unbiased screen for increased susceptibility to infection [66]. It is interesting that both in plants and animals regulation of endogenous genes by RNAi has evolved to promote fitness.

## Materials and Methods

### *C. elegans* mutant and transgenic strains

Strains were maintained at 20°C unless otherwise noted, using standard methods [67]. The following mutants were used: LGI: *daf-16(mu86)*, *daf-16(mgDf50),* LGII: *age-1(hx546),* LGIII: *daf-2(e1370)*, *rde-4*(*ne299), zfp-1(ok554),* LGX: *pdk-1(sa709).*

Compound mutant strains and transgenes used are as follows:

CF1595: *daf-16(mu86)*I*; daf-2(e1370)*III, AGK138: *zfp-1(ok554)*III; *pdk-1(sa709)*X, AGK241: *rde-4(ne299)*III; *pdk-1(sa709)*X, AGK25: *age-1(hx546)*II; *zfp-1(ok554)*III, AGK264: *age-1(hx546)*II; *rde-4(ne299)*III, AGK72: *daf-16(mgDf50)*I; armEx5, TJ356: zIs356 IV, AGK30: *zfp-1(ok554)*III; zIs356 IV, AGK262: *zfp-1(ok554)*III; zIs356 IV; *pdk-1(sa709)*X, AGK377: *rde-4(ne299)*III; zIs356 IV, AGK 265: *rde-4(ne299)*III; zIs356 IV; *pdk-1(sa709)*X, AGK267: *zfp-1(ok554) unc-119(ed3)*III; armIs5, AGK248: *rde-4(ne299) zfp-1(ok554) unc-119(ed3)*III; armIs5, AGK260: zIs356 IV; *pdk-1(sa709)*X, SP940: *unc-52(e444)*II; *unc-1(e538)*X; mnDp11(II;X;f).

Transgenic worms were created by microparticle bombardment using a PDS-1000 Hepta Apparatus (Bio-Rad) [68]. All strains were made by co-bombardment of both a fosmid of interest and plasmid pMM016b (AddGene) for *unc-119(ed3)*III rescue. Strains created are as follows: AGK29: armIs2 Is[unc-119+] – control strain, AGK128: armIs5 Is[ZFP-1::FLAG,unc-119+], AGK26: armEx5 Ex[ZFP-1::GFP,unc-119+].

### Recombinant fosmid construction

The WRM0629bD09 fosmid containing the ZFP-1 locus was obtained from the *C. elegans* fosmid library generated by *C. elegans* Reverse Genetics Core Facility, Vancouver, B.C., Canada.

http://www.lifesciences.sourcebioscience.com/clone-products/genomic-dna-clones.aspx

We generated derivative fosmid constructs to express recombinant ZFP-1 protein tagged with GFP or FLAG at the C-terminal portion of the protein by a fosmid recombineering method as described by [34].

### Oxidative stress assays (paraquat sensitivity)

Paraquat sensitivity assays were done essentially as described by [69]. L4 animals were transferred from NGM agar plates into 24-well plates (10 per well) containing 300 µL of 100 mM paraquat dissolved in M9. Worms were then incubated at 20°C and scored for survival after 20 hours. Dead animals were scored by their continuous absence of swimming movements and pharyngeal pumping. A t-test between two means was used to calculate statistical significance.

### Lifespan assays

Assays were performed as described by [70]. Worms were kept at 20°C on NGM plates (10 animals per plate). Day of hatching was used as the first time point. Dead animals were scored as dead when they refused to move after repeated prodding with a pick. Animals that crawled away from the plate, exploded, or contained internally hatched worms were excluded from the analysis. Life spans were determined in parallel for all strains shown together on graphs. Statistical significance was determined by a log-rank analysis using Prizm software.

### *P. aeruginosa* infection

*C. elegans* survival assays were performed as described earlier [36]. To avoid the confounding effects of varying brood sizes, egg laying rates and progeny hatching within the infected worms on worm mortality, we used worms rendered sterile by RNAi of *pos-1*, loss of which results in inviable embryos [71], [72]. Worms that died due to desiccation on the walls of the Petri dish or due to bursting vulva were censored from further analysis. Statistical analysis was performed using Kaplan-Meier non-parametric survival analysis using the software Statview (Version 5.0.1 SAS Institute Inc.). *P*<0.001 was considered significantly different than wild type.

### Analysis of the nuclear localization of DAF-16::GFP

Since the addition of the DAF-16::GFP transgene to the *zfp-1(ok554); pdk-1(sa709)* double mutant strain led to a penetrant dauer phenotype at 20°C, all DAF-16::GFP strains were maintained at 16°C. L4 and adult stage worms were used for scoring nuclear localization. Worms were mounted on agarose pads and DAF-16::GFP localization was assessed in 10–20 worms at a time using 200X magnification on a Zeiss AxioImager Z1 immediately, higher magnification images of DAF-16::GFP localization in intestinal cells were done at 630X.

### RNA extraction and RT-qPCR

Synchronous populations of animals were grown at 20°C on NGM plates seeded with OP50 *E. coli* at a density of approximately 100,000 animals per 15 cm Petri dish and harvested at specific stages of development. The harvested animals were washed three times with M9 buffer and the pellet was frozen in dry ice with TRI Reagent (MRC, Inc.). After five times of freeze and thaw, total RNA was isolated according to the TRI Reagent protocol. Ten micrograms of the total RNA sample was digested with 2U of Turbo DNase (Ambion) at 37°C for 1hr followed by phenol-extraction and ethanol-precipitation. cDNA was generated from 2 µg of total RNA, using oligo-dT primer and RevertAid Reverse Transcriptase (Fermentas). Quantitative PCR was performed on the Mastercycler ep realplex (Eppendorf) using the QuantiFast SYBR Green PCR Kit (Qiagen). Thermocycling was done for 40 cycles in a two-step cycling, according to the manufacturer's instructions, with 25 µl of reaction containing 12.5 µl SYBR master mix, 0.15 µl of 100 µM primers, 5 µl of diluted cDNA, and 7.2 µl dH2O. Each PCR reaction was performed in triplicate. We used the ΔΔCt method to quantify the change in mRNA expression in the mutant samples compared to wild type and *act-3* mRNA was used as a reference gene. The primers used were as follows: Forward CACGAGACTTCTTACAACTCC and Reverse GCATACGATCAGCAATTCCT for *act-3* mRNA detection, Forward AGCCATCAACACCGTCTAAC and Reverse CGAATTGGCGCGTGGTGC for *pdk-1* mRNA detection, Forward GCTAGGATGTCAGGTGGTC and Reverse CCAAGAGAAGCCACGAAAGC for *aqp-1* mRNA detection, Forward ATGCTCGTGCTCTTGCTGAG and Reverse GACTGACCGAATTGTTCTCCAT for *gst-4* mRNA detection, Forward TACCGATGAGGAGTGGGAGA and Reverse CGAATTCCCGAGCAAGATAA for *gst-38* mRNA detection, Forward TTTCAGAATCACAGAGCAACAC and Reverse TGCGATACATGTTCAGAAGAG for *zfp-1* mRNA detection, Forward ACACTATTAAGCGCGACTTCG and Reverse AGTTGGCAATCTTCCAAATAGC for *sod-3* mRNA detection, Forward *pdk-1* ex2-ex3 junction CCTACAGCCAGGTATTCCG and Reverse *pdk-1* intron 3 ACAAGTGGATTTTGATGGGTTC for detecting the mutant *sa709 pdk-1* mRNA and pre-mRNA and Reverse *pdk-1* ex3-4 junction GATCACGAAATAAATTCTAGCCTGG-for detecting the wild-type *pdk-1* mRNA.

For detection of bi-directional transcription at the *pdk-1* promoter the primers used were as follows. Region 1 RT primers: detecting (−) strand transcript CCGAGGTTATAATTTTGGCTAAACTT; detecting (+) strand transcript ATCAAGAGATACAGCGGGAG. Region 1 PCR primers:

forward- CGGAGTTATAACCAAGCAACCA

reverse- GTGTCAACTGGATATGAATCCGAA

Region 2 RT primers: detecting (−) strand transcript CTCCCGCTGTATCTCTTGAT

detecting (+) strand transcript GTACGGTTGTTATCGCTTTCAGG.

Region 2 PCR primers: forward - GAATGTTCAAAGCCTTAAAGC

reverse – AGGGATAATTGGAGTGACATGG.

### Chromatin immunoprecipitation (ChIP)

Chromatin immunoprecipitation was performed following the modENCODE Protocol from the Lieb Lab with the following modification: 2.5–3mg of cross-linked extract from L3 or adult worms was incubated for 1h at 4°C with the specific antibody and the immune complexes were then incubated with 60 µl IgG Dynabeads (Invitrogen) for 1h at 4°C. DNA was cleaned up with the Qiagen PCR purification kit. For the FLAG ChIP, we incubated the cross-linked extract with ANTI-FLAG M2 Affinity Gel (Sigma) for 2h at 4°C and, after the washing steps, eluted with 300 µg/ml of FLAG peptide (Sigma) for 30min at 4°C. The other antibodies used were anti-ZFP-1 (generated by the Lieb Lab) and anti-Pol II 8WG16 (Covance).

The immunoprecipitated DNA was quantified by qPCR using the ΔΔCt method to calculate the percentage of immunoprecipitation relative to the input. We used the following specific primers: Forward AAACAACACATAGACTTGTGCC and Reverse GTACGGTTGTTATCGCTTTCAG to amplify the promoter region of the *pdk-1* gene; Forward *pdk-1* ex2 GCAAGTGAATCGGAGAACAG and Reverse *pdk-1* ex2 TGAAGAAACATGAAGTGCTTGG to amplify the coding region of the *pdk-1* gene; Forward TTTCAGAACTATCATGCCACG and Reverse TCTCTGAGCACACTTTGAGG to amplify the promoter region of the *aqp-1* gene; Forward *aqp-1* ex5 TTGCCAGTTATCCATCTCCA and Reverse *aqp-1* ex5 CTCTCATCAATAACAACGCAG to amplify the coding region of the *aqp-1* gene; Forward TTAGATAGAGAATTGGCGAGAG and Reverse CAAGTAGCAAAGCGATAAACC to amplify the promoter region of the *gst-4* gene; Forward *gst-4* ex4 TGAAGTTGTTGAACCAGCC and Reverse *gst-4* ex4 CCCAAGTCAATGAGTCTCCA to amplify the coding region of the *gst-4* gene.

### modENCODE protocols

To investigate the function of ZFP-1 with ChIP we first developed an antibody (termed JL00006_ZFP1) to the C-terminal portion of the protein. Alternative transcription start sites give rise to two ZFP-1 protein isoforms with identical C-terminal domains. As expected, both isoforms are recognized by the JL00006_ZFP1 antibody. The protocols used for generating ZFP-1 ChIP/chip data are described at http://www.modencode.org/Lieb.shtml.

### Determining genes bound by ZFP-1

*C. elegans* genes (refSeq id) from genome build CE4 (ws170) were extracted from the UCSC genome browser's refGene table. A gene was called bound by ZFP-1 if the center base pair of a ZFP-1 peak overlapped the ORF or the 1,500 bp upstream region. Overlap calls were done using the Galaxy web tool. Of the total 24,901 genes, 3,598 were bound by ZFP-1. Genome-wide ZFP-1 localization data are available at modENCODE: http://intermine.modencode.org/.

## Supporting Information

Figure S1 Increase in *pdk-1* expression correlates with susceptibility to oxidative stress. (A, B) Survival of L4 larva (n = 90) from indicated strains after 20 hour incubation period in 100mM paraquat; ** indicates significance of P<0.01 and * - P<0.05 compared to wild type. (C) RT-qPCR detecting an increase in *pdk-1* expression in SP940, results of three biological replicas are shown, error bars represent standard deviation.
(TIF)

Figure S2 RT-qPCR confirming enhanced expression of *zfp-1* mRNA in ZFP-1::FLAG and ZFP-1::GFP transgenic strains.
(TIF)

Figure S3 ZFP-1 levels do not change in *rde-4(ne299).* Western blot analysis with anti-ZFP-1 C-terminal antibody; actin levels and Ponceau S staining of the membrane are shown as loading controls.
(TIF)

Figure S4 An example of a gene with promoter siRNAs matching repetitive elements and expressed higher in *rde-4(ne299)* according to [19]. A screen shot from the UCSC browser and PolII ChIP results showing enhanced occupancy in *rde-4(ne299)*.
(TIF)

Figure S5 Examples of genes with promoter siRNAs matching repetitive elements and expressed higher in *rde-4(ne299)* according to [19]. Screen shots from the UCSC browser.
(TIF)

Table S1 Dataset table allowing identification of overlaps between the following datasets: microarray data listing genes misregulated in *rde-4(ne299)* and *zfp-1(ok554)* from [19], microarray data of Class 1 and Class 2 genes acting downstream of *daf-16* from [23], *rde-4*-dependent siRNA target genes identified by [38], and ZFP-1 target genes where the ZFP-1 peak was found in the 1,500bp promoter window identified by ChIP/chip; functional mount and category groups from [73].
(XLSX)

Text S1 Sequencing results for RNA sequences produced from the *pdk-1* promoter and shown in Figure 7D.
(DOC)
