# Supplementary figures and images for: A Conserved PHD Finger Protein and Endogenous RNAi Modulate Insulin Signaling in Caenorhabditis elegans
Source: PLoS Genet. 2011 Sep 29;7(9):e1002299. doi: 10.1371/journal.pgen.1002299 (PMC3183084; doi:10.1371/journal.pgen.1002299)

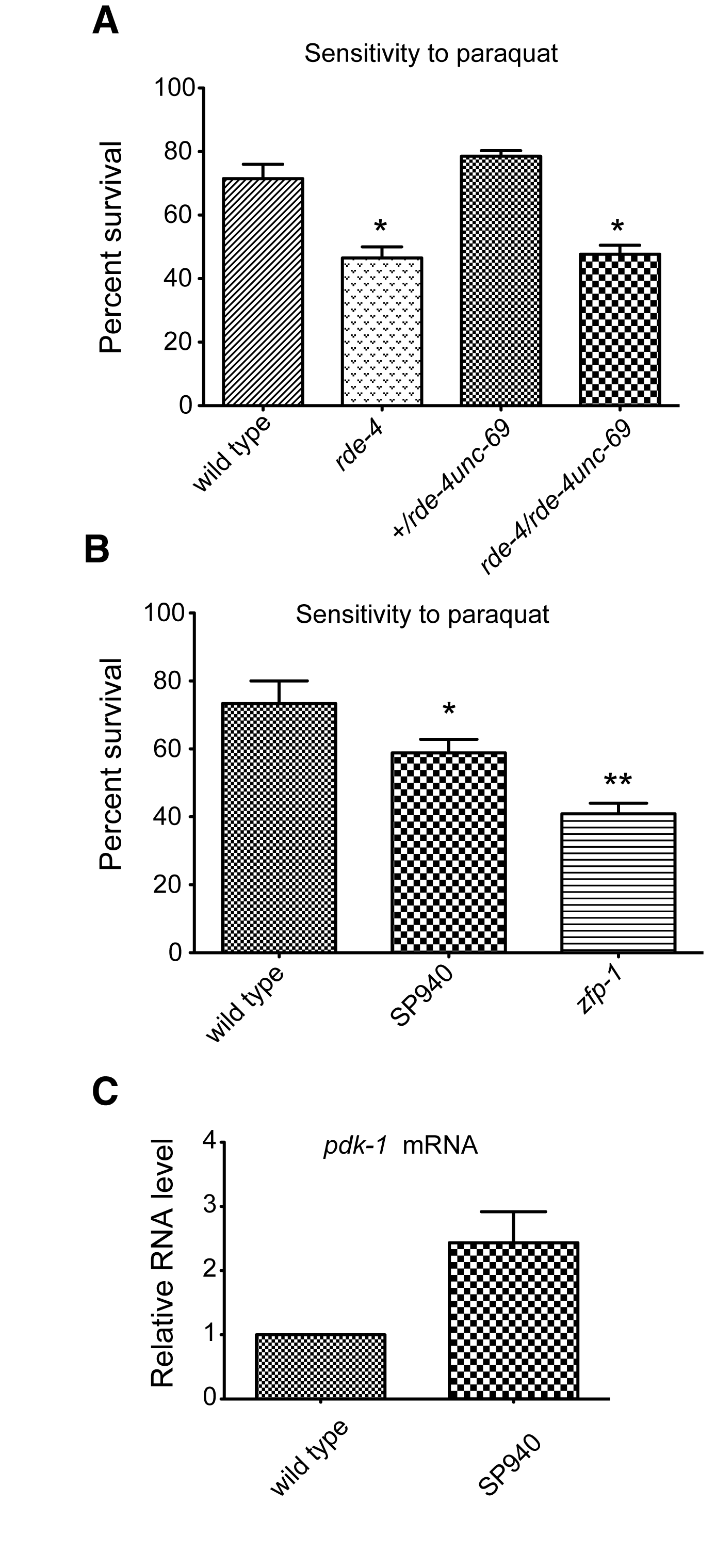

Supplement: Figure S1 — Increase in pdk-1 expression correlates with susceptibility to oxidative stress. (A, B) Survival of L4 larva (n = 90) from indicated strains after 20 hour incubation period in 100mM paraquat; ** indicates significance of P<0.01 and * - P<0.05 compared to wild type. (C) RT-qPCR detecting an increase in pdk-1 expression in SP940, results of three biological replicas are shown, error bars represent standard deviation. (TIF) [file pgen.1002299.s001.tif]

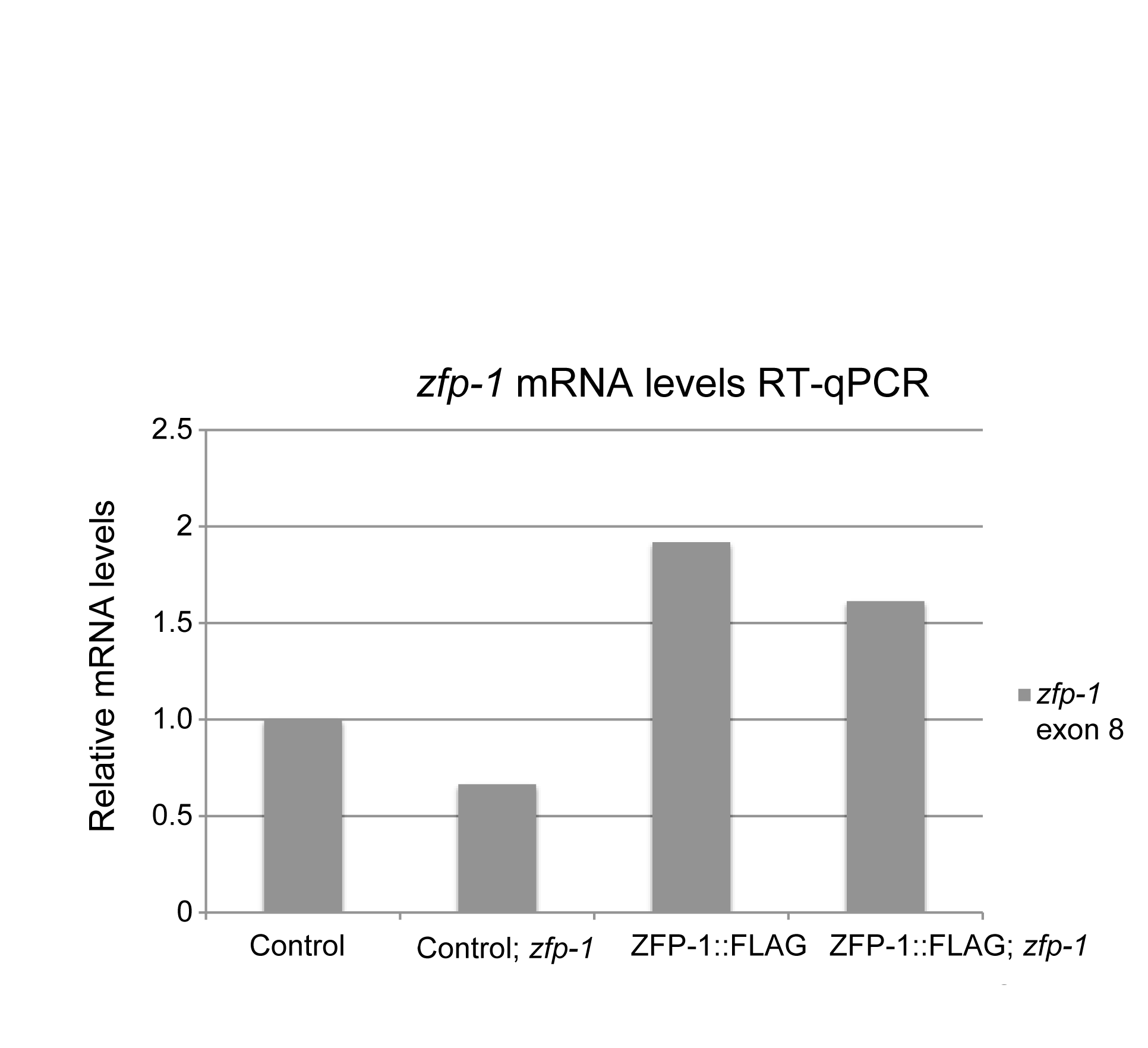

Supplement: Figure S2 — RT-qPCR confirming enhanced expression of zfp-1 mRNA in ZFP-1::FLAG and ZFP-1::GFP transgenic strains. (TIF) [file pgen.1002299.s002.tif]

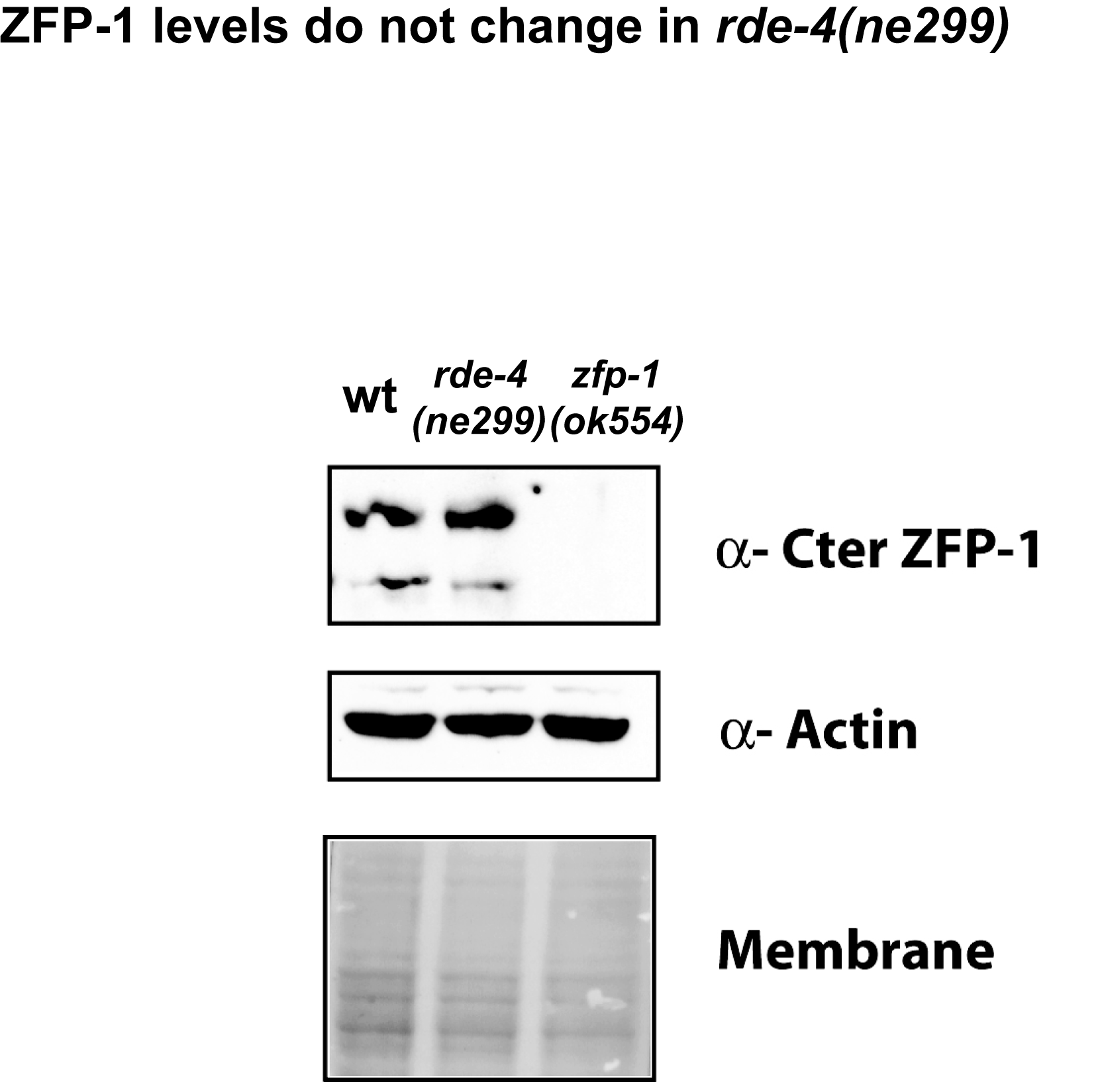

Supplement: Figure S3 — ZFP-1 levels do not change in rde-4(ne299). Western blot analysis with anti-ZFP-1 C-terminal antibody; actin levels and Ponceau S staining of the membrane are shown as loading controls. (TIF) [file pgen.1002299.s003.tif]

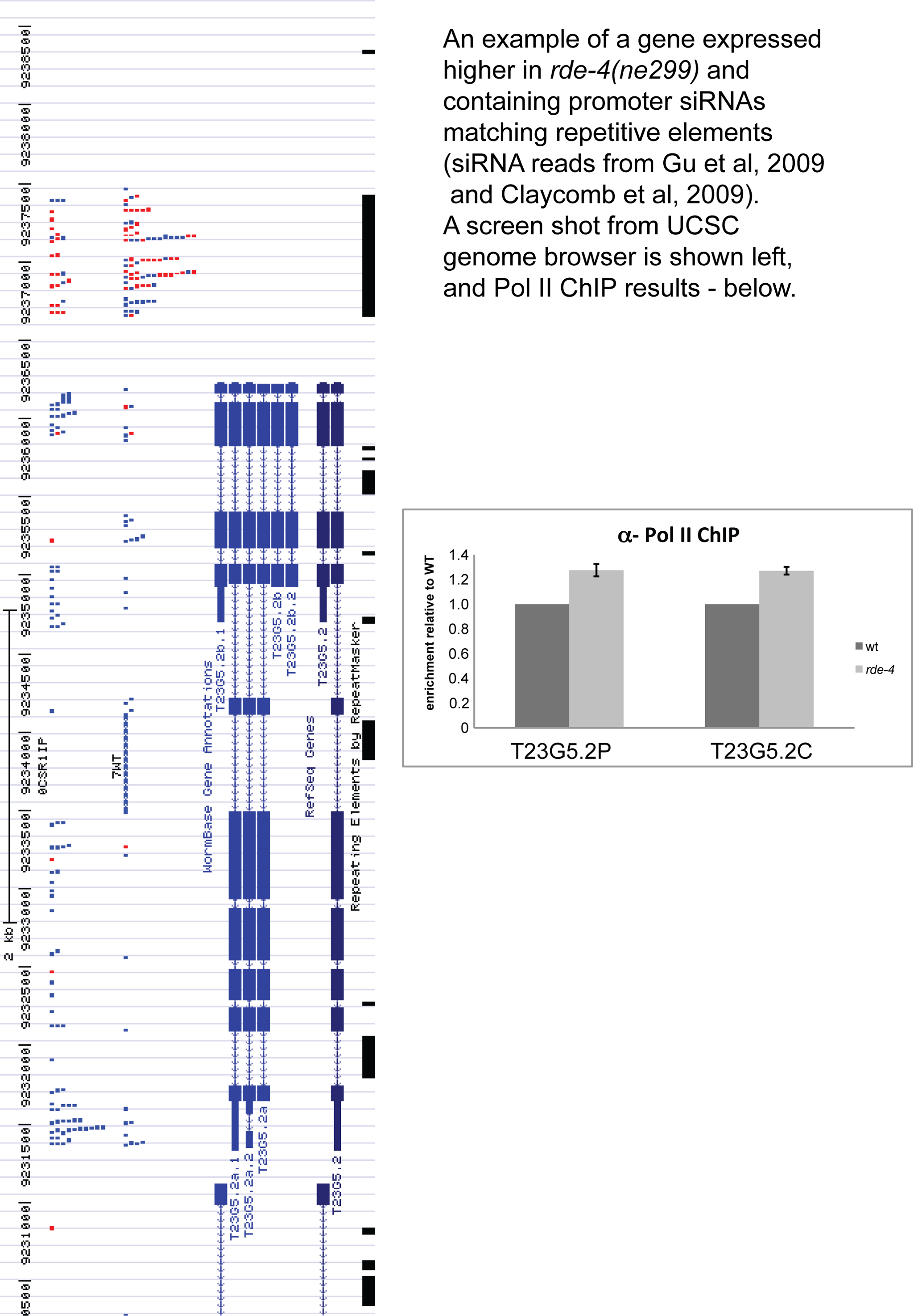

Supplement: Figure S4 — An example of a gene with promoter siRNAs matching repetitive elements and expressed higher in rde-4(ne299) according to [19]. A screen shot from the UCSC browser and PolII ChIP results showing enhanced occupancy in rde-4(ne299). (TIF) [file pgen.1002299.s004.tif]

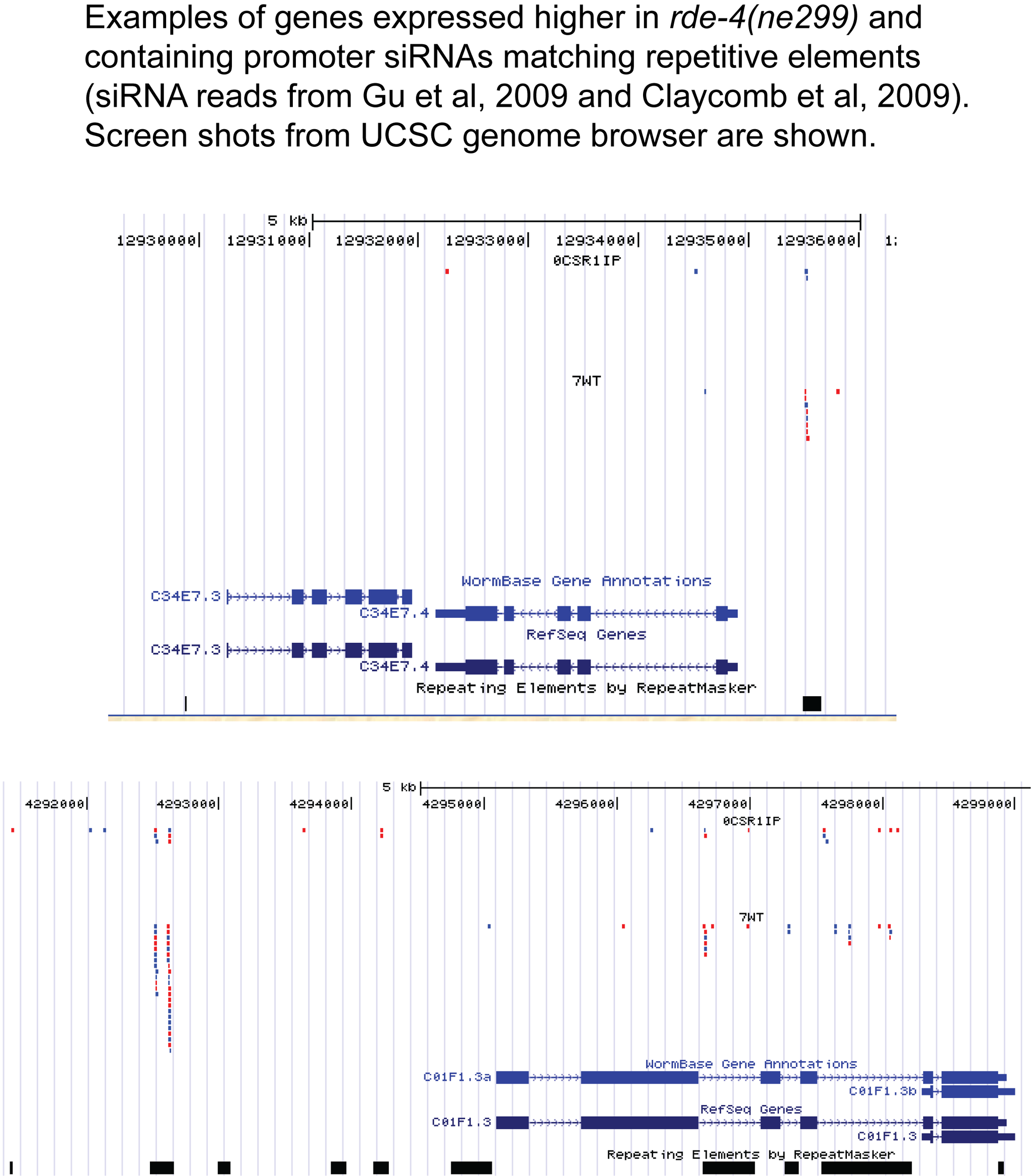

Supplement: Figure S5 — Examples of genes with promoter siRNAs matching repetitive elements and expressed higher in rde-4(ne299) according to [19]. Screen shots from the UCSC browser. (TIF) [file pgen.1002299.s005.tif]
