# Supplementary material for: A Conserved PHD Finger Protein and Endogenous RNAi Modulate Insulin Signaling in Caenorhabditis elegans
Source: PLoS Genet. 2011 Sep 29;7(9):e1002299. doi: 10.1371/journal.pgen.1002299 (PMC3183084; doi:10.1371/journal.pgen.1002299)
Supplement: Text S1 — Sequencing results for RNA sequences produced from the pdk-1 promoter and shown in Figure 7D. (DOC) [file pgen.1002299.s007.doc]

**Text S1**

**Sequencing results for RNA sequences produced from the *pdk-1* promoter and shown in Figure 7 and below.**


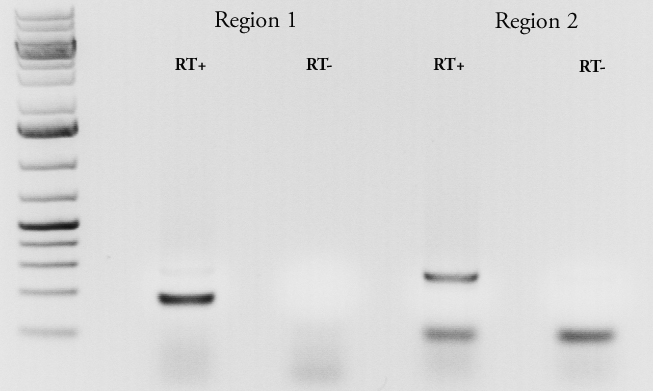


RT was done with random hexamer primers.

**Region 1 of the *pdk-1* promoter**

Sequencing primer: ccaagcaaccacgtaataata

**Sequencing result:**

CTTTTTTTTT**A**ACTTACAATGTTCTATACTTTTAGTTTTTGACGTTATTTGTGCTTTTGGCTAATACACTTTTTGATTAAAATTTTTTTCGGATTCATATCCAGTTG

***pdk-1* promoter region 1 (from the genome browser):**

>X:1318400..1318799

Gttgcccatgtcttgccagaaacttctaaaccgaggttataattttggctaaactttgccataaagttcgtaccagctaaacatttgttttattttttaacgtgtttttaggaaaaaaaacaatattttatattttaattgacgtaatcaaatttttttacctgtcattttttccaatatatagggt**cggagttataaccaagcaacca**cgtaataataatattaatttttaaaaaactttttttttaacttacaatgttctatacttttagtttttgacgttatttgtgcttttggctaatacactttttgattaaaattttt**ttcggattcatatccagttgacac**ttaattctctaaaaacttttttttaacttggccatttttgtatattttaaac

Underlined are primer sequences used to amplify region 1 in the RT-PCR.

Red - the primer used to sequence.

Yellow - the sequencing result.

**Region 2 of the *pdk-1* promoter**

Sequencing primer: caaagccttaaagcctaacatg

**Sequencing result:**

AATGGTTACA**A**TTATTTATTTTTTCAGTTTGCTTTTACTGACAAATGTATTTTTTTGATAATAAACAAAAAAAACATAGAAAGTTGCAATTTTGGACATTTTTTCTATTTTTTCACATTTTTCAGTGTTATTGACCGAGCTGTAAGTAATCCCAGCACATTTTCCATGTCACTCCAATTATCCC

***pdk-1* promoter region 2 (from the genome browser):**

>X:1319100..1319699

gctgtatctcttgatctgaattttttgaaatgttgaatcaagtgttaacaggtagacaagattctaaacaacagtttatctgttaatttttttttctaaaatgcttagtttgtgagttatactcatttgaaagttataaagaattagcaaaaattgtaatttttcaaccgaatcagaacatgttt**gaatgttcaaagccttaaagc**ctaacatgtaaaattaaaaaatatgaaatggttacaattatttattttttcagtttgcttttactgacaaatgtatttttttgataataaacaaaaaaaacatagaaagttgcaattttggacattttttctattttttcacatttttcagtgttattgaccgagctgtaagtaatcccagcacatttt**ccatgtcactccaattatccct**ggcgcttgctttcagtctcaattttcccaaaactatttcttcaaaaacatttccaaaggcgccccttgtgtcaactgcaaacactcgccccgatgacgtaacttgtttacgacgaaaaaaaaagtgtttcttcgtggtttataactcaacttgttgatttcattttagttccgggaaacagaa

Underlined are primer sequences used to amplify region 2 in the RT-PCR.

Red - the primer used to sequence.

Yellow - the sequencing result.
